# Supplementary figures and images for: The Effect of Macromolecular Crowding, Ionic Strength and Calcium Binding on Calmodulin Dynamics
Source: PLoS Comput Biol. 2011 Jul 28;7(7):e1002114. doi: 10.1371/journal.pcbi.1002114 (PMC3145654; doi:10.1371/journal.pcbi.1002114)

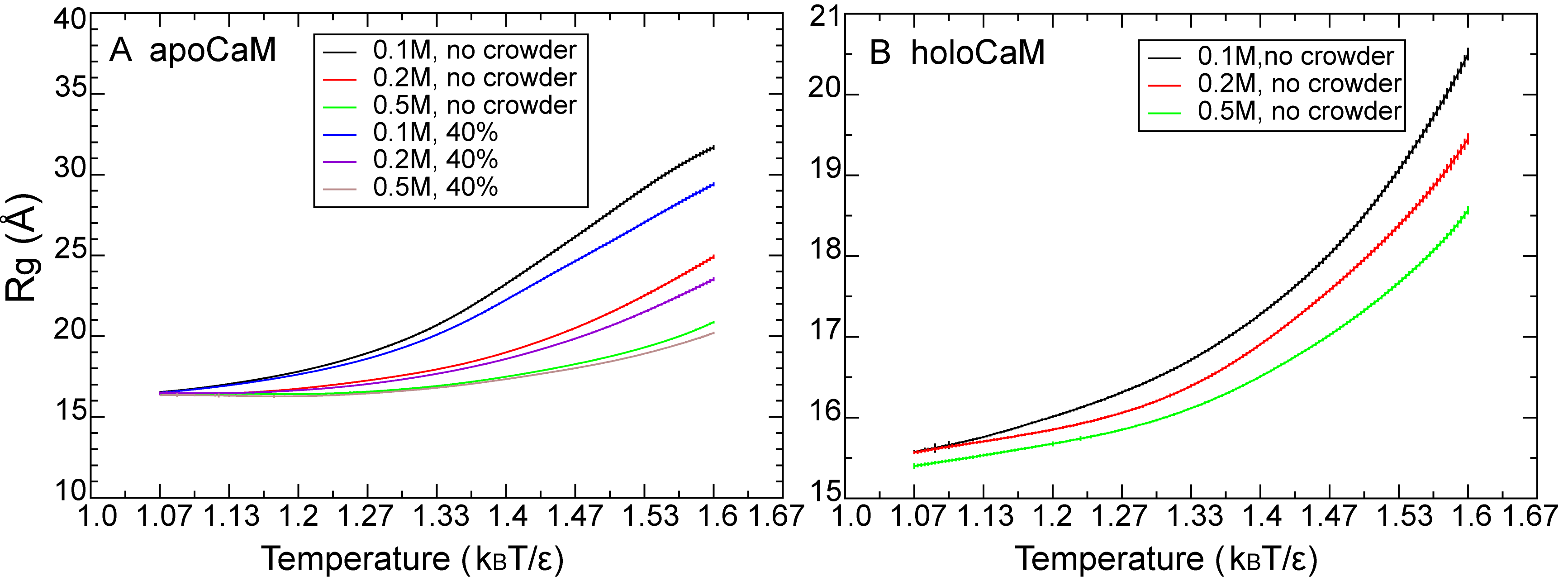

Supplement: Figure S1 — Radius of gyration (Rg) of (A) apoCaM and (B) holoCaM as a function of temperature in solutions with various ionic strengths. Error bars (very small) are included. (TIF) [file pcbi.1002114.s001.tif]

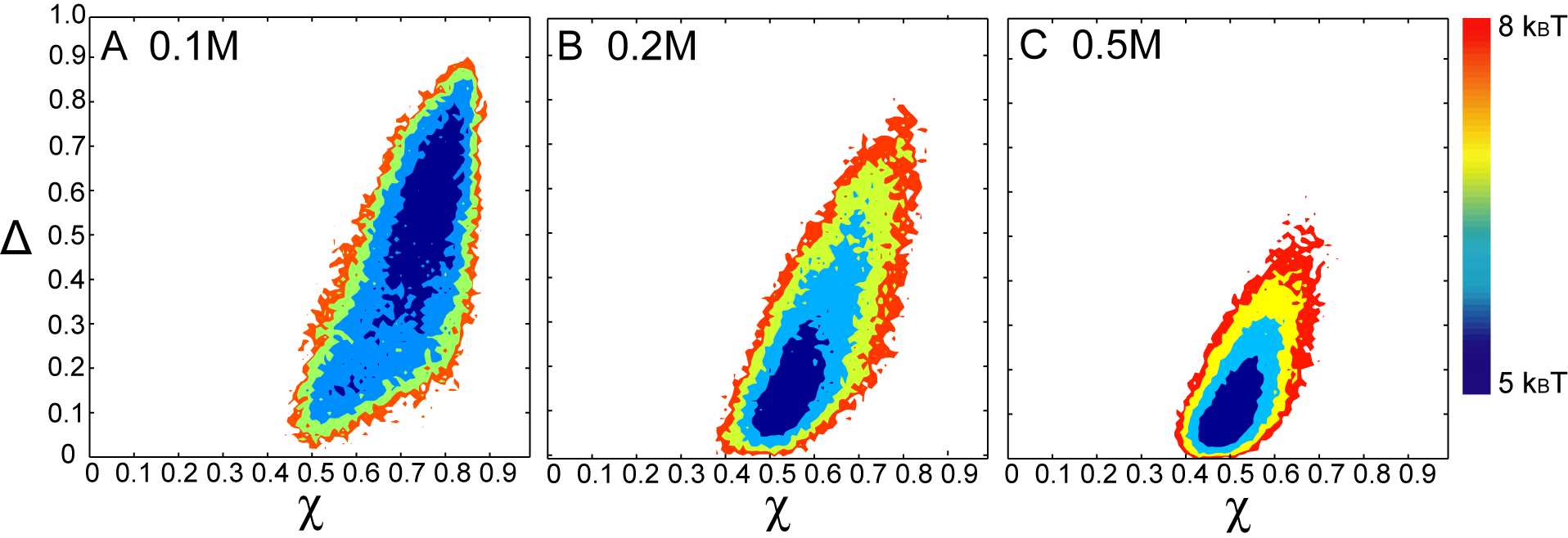

Supplement: Figure S2 — 2-D free energy landscape of apoCaM as a function of overlap function (χ) and asphericity (Δ) at different ionic strengths at 1.5 kBT/ε. (A) [KCl] = 0.1 M, (B) [KCl] = 0.2 M and (C) [KCl] = 0.5 M. (TIF) [file pcbi.1002114.s002.tif]

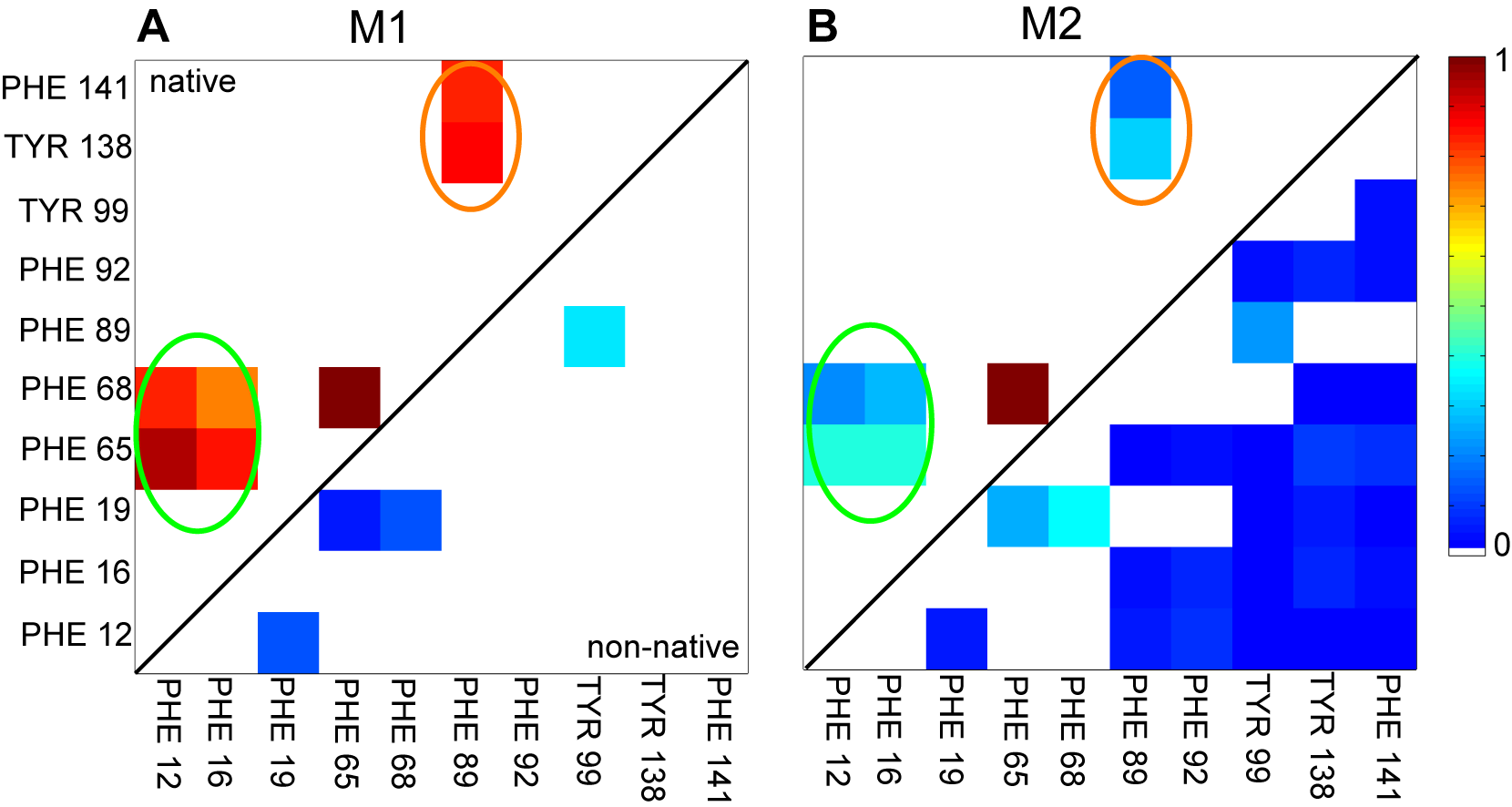

Supplement: Figure S3 — Probability of contact formation between phenylalanine and tyrosine in different ensemble conformations of apoCaM. (A) M1 and (B) M2 at T = 1.15 kBT/ε. (TIF) [file pcbi.1002114.s003.tif]

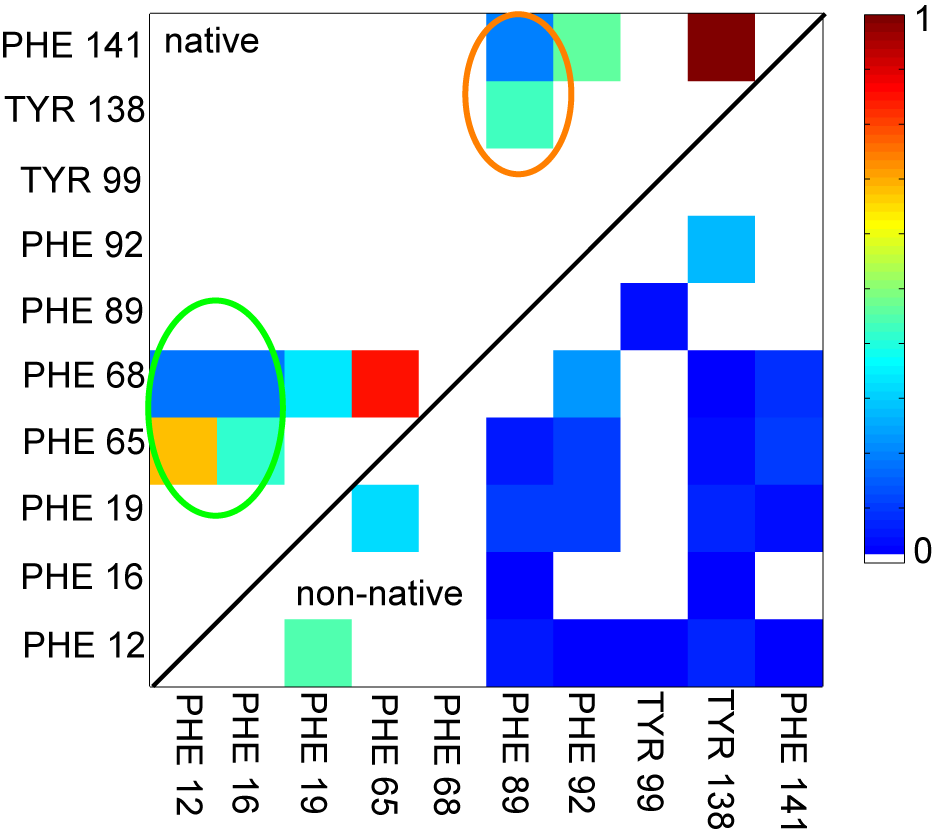

Supplement: Figure S4 — Probability of contact formation between phenylalanine and tyrosine for the M3 state of holoCaM. T = 1.15 kBT/ε. Color bar ranges from blue (0) to red (1). (TIF) [file pcbi.1002114.s004.tif]

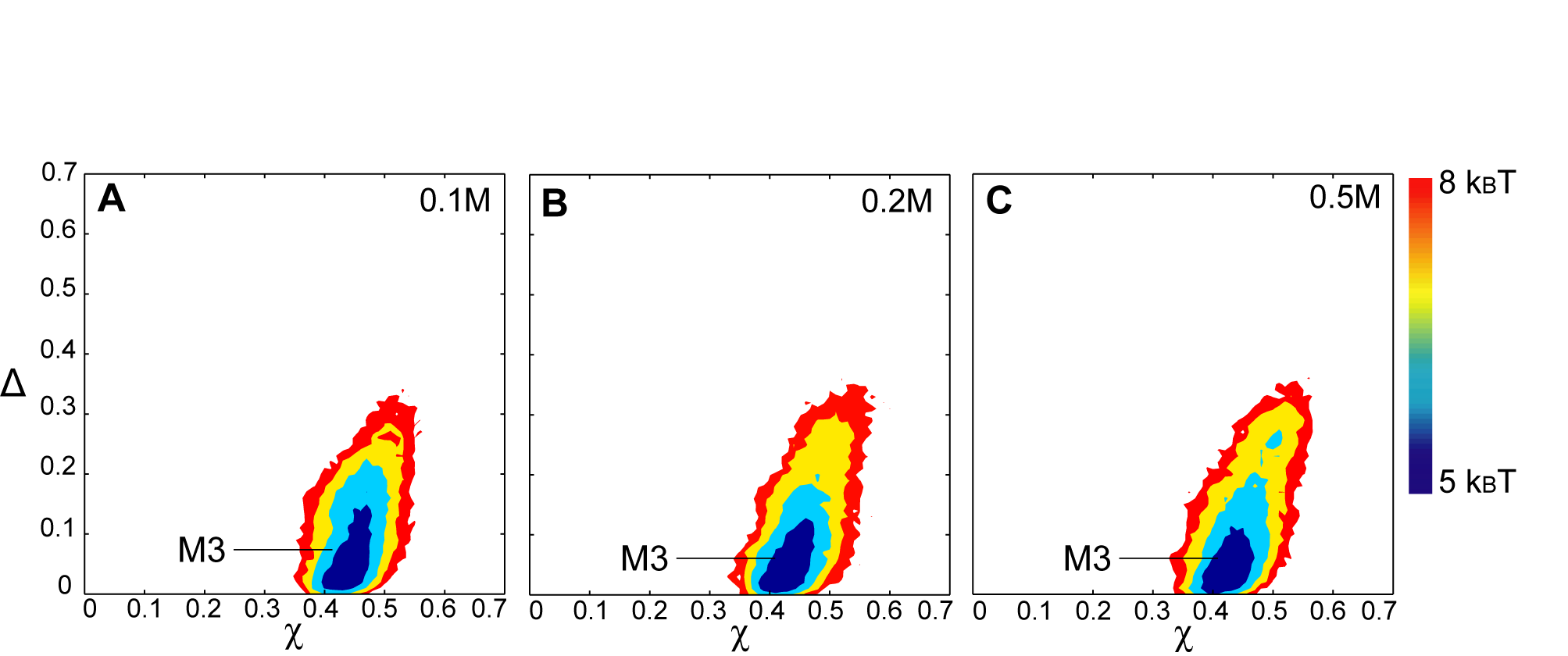

Supplement: Figure S5 — 2-D free energy landscape of holoCaM in different ionic strengths at T = 1.15 kBT/ε. X axis is the overlap function (χ) and Y axis is the asphericity (Δ). (A) [KCl] = 0.1 M. (B) [KCl] = 0.2 M. (C) [KCl] = 0.5 M. (TIF) [file pcbi.1002114.s005.tif]

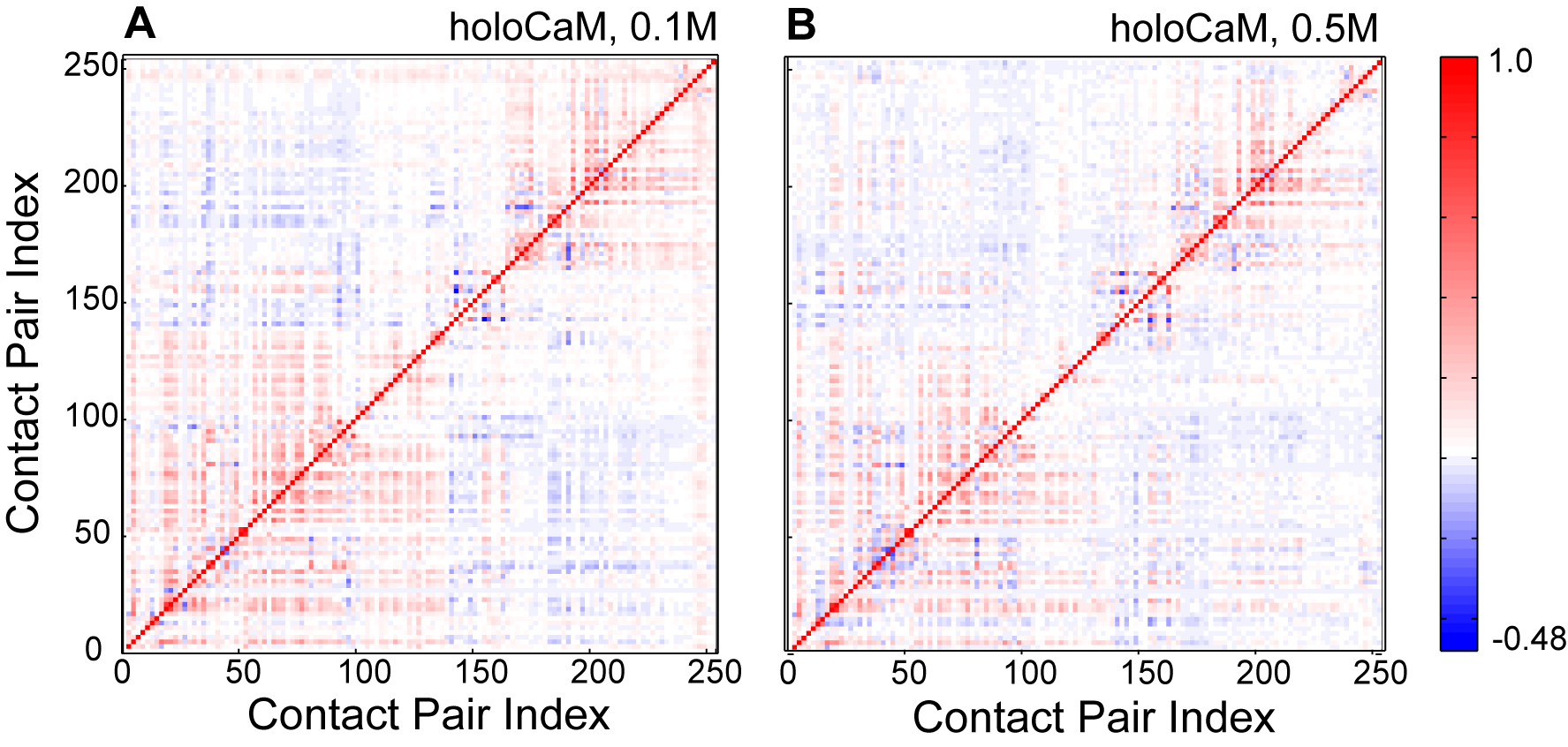

Supplement: Figure S6 — Covariance matrix of contact formation of holoCaM. The covariance matrix is plotted as a function of Contact Index Pairs (see Table S4 in Text S1) at different ionic strengths at 1.15 kBT/ε. (A) [KCl] = 0.1 M. (B) [KCl] = 0.5 M. (TIF) [file pcbi.1002114.s006.tif]

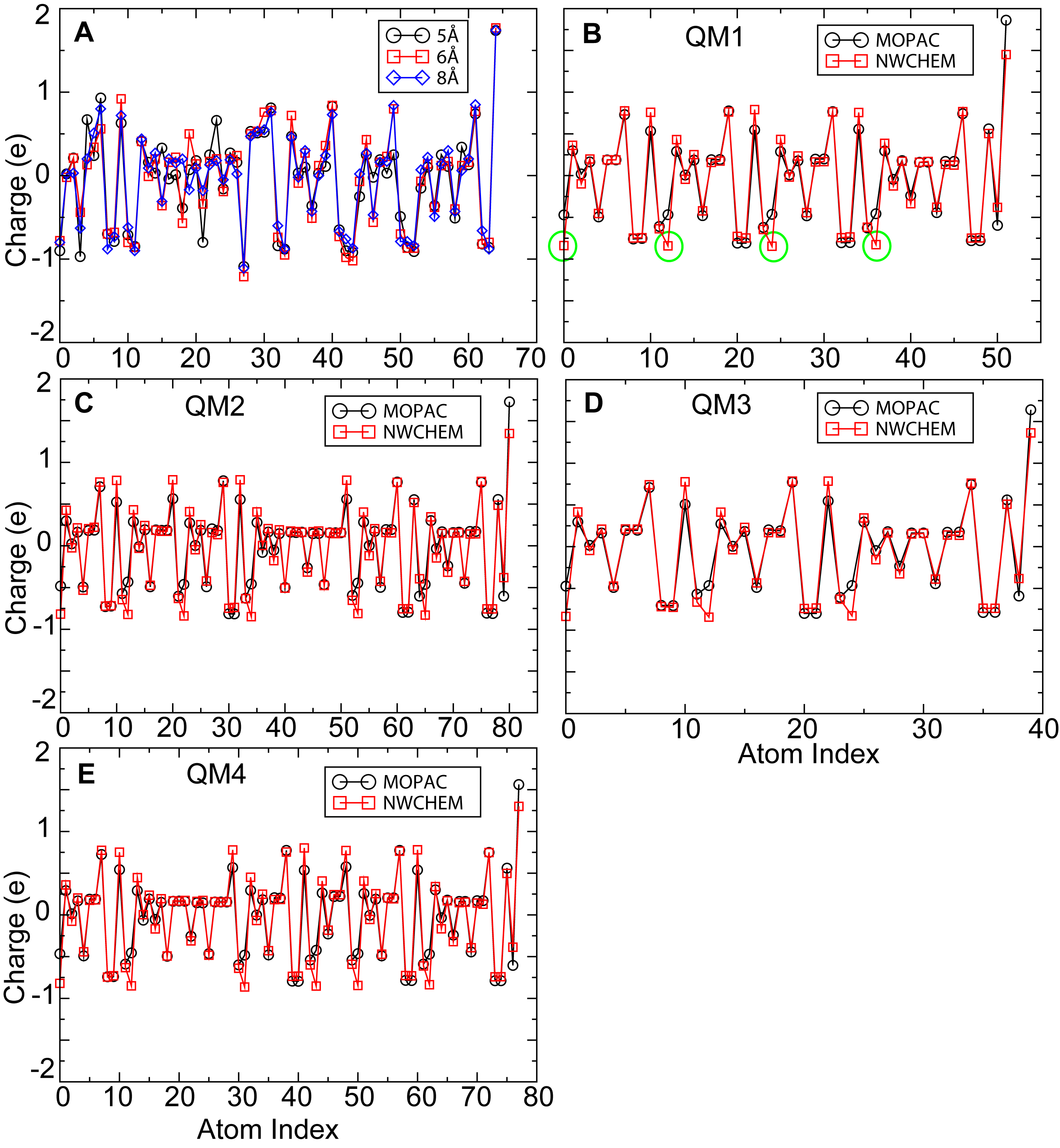

Supplement: Figure S7 — Comparison of the atomic charges calculated by AM1 semi-empirical (MOPAC) and ab initio (HF/6-31G*) QM/MM (NWCHEM) methods. (A) Atomic charges calculated with different sizes of quantum mechanical (QM) region. (B) Atomic charges in the QM1 region. (C) Atomic charges in the QM2 region. (D) Atomic charges in the QM3 region. (E) Atomic charges in the QM4 region. Error bars (error of the mean) are smaller than the size of the symbols in the graphs. (See the definition of QM1, QM2, QM3, and QM4 in the Supplement). (TIF) [file pcbi.1002114.s007.tif]
