# Supplementary material for: The Effect of Macromolecular Crowding, Ionic Strength and Calcium Binding on Calmodulin Dynamics
Source: PLoS Comput Biol. 2011 Jul 28;7(7):e1002114. doi: 10.1371/journal.pcbi.1002114 (PMC3145654; doi:10.1371/journal.pcbi.1002114)
Supplement: Text S1 — Comparison of charges based on semi-empirical AM1 (MOPAC) and ab-initio Hartree-Fock/6-31G(d) QM/MM methods. Table S1. Charge distribution on a coarse-grained Cα-side chain model of apoCaM. The unit of charge is 1.6*10−19 C. Table S2. Charge distribution on a coarse-grained Cα-side chain model of holoCaM. The unit of charge is 1.6*10−19 C. Table S3. Contact pair index (CPI) of apoCaM. “b” denotes contacts between side chain beads and “h” denotes contacts between Cα beads. Table S4. Contact pair index (CPI) of holoCaM. “b” denotes contacts between side chain beads and “h” denotes contacts between Cα beads. (DOC) [file pcbi.1002114.s008.doc]

Text S1

Comparison of charges based on semi-empirical AM1 (MOPAC) and ab-initio Hartree-Fock/6-31G(d) QM/MM methods: To obtain the partial atomic charges we used the semi-empirical AM1 charge model as implemented in MOPAC . In order to verify the quality of the atomic charges calculated by MOPAC, the Mulliken atomic charges of holoCaM were calculated at the Hartree-Fock (HF) level with 6-31G(d) basis set using NWCHEM 6.0. Due to the size of holoCaM, the Hartree-Fock calculation on the whole structure is not feasible. Instead, we defined a quantum mechanical (QM) region that includes all residues within 6 Å of each of the four calcium ions. In order to examine the effect of the size of the QM region we have tested the QM regions within 5, 6, and 8 Å radius around calcium. The inspection of the calculated charges shows a good agreement between charges calculated for the QM regions with 6 and 8 Å radius (Figure S7A). Because the HF calculations are already computationally quite intensive for the QM region with 6 Å radius and the time required to complete the calculations increases by the factor of 2.2 when the size of the QM is increased from 6 to 8 Å, we adopted a QM region within 6 Å radius around a calcium. There are four QM regions for each configuration of holoCaM: QM1, QM2, QM3 and QM4 containing all residues with 6 Å of the first, second, third and fourth calcium in a structure. Each configuration of CaM was solvated with an extra 5-Å pad of TIP3P water model in a cubic box. The system was energetically minimized using CHARMM22 with fixed atoms of CaM Any solvent molecules present within 6 Å radius around the Ca atoms were also added explicitly into the QM region. The N- and C-terminus of a residue selected in the QM region were individually acetylated and capped by COOH, respectively. All atoms of a protein outside the QM region were represented as point charges (computing from MOPAC). The HF calculations were subsequently carried out for each QM region of the 400 configurations of holoCaM and obtained Mulliken charges for each atom in the QM region. The charge of each atom was then averaged over all 400 configurations. Figure S7 (B to E) shows the comparison between MOPAC calculation and the QM/MM calculations based on Hartree-Fock in four QM regions. Most charges calculated by two methods match fairly well (the difference is smaller than 0.1e), indicating that the charges used in our simulations were reasonable. There are few atoms (e.g. circled in green in Figure S7B) that have relatively large difference between MOPAC calculation and Hartree-Fock calculation (the difference is 0.4e). This difference arises from the fact that when we capped the selected residues in the QM region, the chemistry of a bond was altered at the terminus of those residues.

CAPTIONS

Figure S1. Radius of gyration (R_g_) of (A) apoCaM and (B) holoCaM as a function of temperature in solutions with various ionic strengths. Error bars (very small) are included.

Figure S2. 2-D free energy landscape of apoCaM as a function of overlap function (χ) and asphericity (Δ) at different ionic strengths at 1.5 k_B_T/ε: (A) [KCl]=0.1M, (B) [KCl]=0.2M and (C) [KCl]=0.5M.

Figure S3. Probability of contact formation between phenylalanine and tyrosine in different ensemble conformations of apoCaM. (A) M1 and (B) M2 at T= 1.15 k_B_T/ε.

Figure S4. Probability of contact formation between phenylalanine and tyrosine for the M3 state of holoCaM. T= 1.15 k_B_T/ε. Color bar ranges from blue (0) to red (1).

Figure S5. 2-D free energy landscape of holoCaM in different ionic strengths at T = 1.15 k_B_T/ε. X axis is the overlap function (χ) and Y axis is the asphericity (Δ). (A) [KCl]=0.1M. (B) [KCl]=0.2M. (C) [KCl]=0.5M.

Figure S6. Covariance matrix of contact formation of holoCaM. The covariance matrix is plotted as a function of Contact Index Pairs (see Table S4) at different ionic strengths at 1.15 k_B_T/ε. (A) [KCl]=0.1M. (B) [KCl]=0.5M.

Figure S7. Comparison of the atomic charges calculated by AM1 semi-empirical (MOPAC) and ab initio (HF/6-31G*) QM/MM (NWCHEM) methods. (A) Atomic charges calculated with different sizes of quantum mechanical (QM) region. (B) Atomic charges in the QM1 region. (C) Atomic charges in the QM2 region. (D) Atomic charges in the QM3 region. (E) Atomic charges in the QM4 region. Error bars (error of the mean) are smaller than the size of the symbols in the graphs. (See the definition of QM1, QM2, QM3, and QM4 in the Supplement)

Table S1. Charge distribution on a coarse-grained C_α_-side chain model of apoCaM. The unit of charge is 1.6*10^-19^ C.

| Residue Index | Residue  Name | Charge (C_α_) | Error (C_α_) | Charge (side chain) | Error  (side chain) |
| --- | --- | --- | --- | --- | --- |
| 1 | ALA | -0.07355 | 0.001472 | 0.045007 | 0.000601 |
| 2 | ASP | -0.02512 | 0.002225 | -0.89938 | 0.002888 |
| 3 | GLN | -0.0577 | 0.002318 | 0.044362 | 0.001058 |
| 4 | LEU | -0.0628 | 0.002485 | 0.077258 | 0.001104 |
| 5 | THR | -0.11185 | 0.002666 | 0.082574 | 0.00124 |
| 6 | GLU | -0.07093 | 0.002257 | -0.86703 | 0.002931 |
| 7 | GLU | -0.07901 | 0.002161 | -0.85989 | 0.002819 |
| 8 | GLN | -0.05683 | 0.002664 | 0.05936 | 0.000974 |
| 9 | ILE | -0.11102 | 0.002668 | 0.113334 | 0.001022 |
| 10 | ALA | -0.05053 | 0.002462 | 0.056385 | 0.000713 |
| 11 | GLU | -0.06765 | 0.002889 | -0.86669 | 0.002785 |
| 12 | PHE | -0.05938 | 0.002629 | 0.054623 | 0.000999 |
| 13 | LYS | -0.09422 | 0.002784 | 1.00845 | 0.00309 |
| 14 | GLU | -0.06704 | 0.002413 | -0.88953 | 0.003003 |
| 15 | ALA | -0.03013 | 0.002475 | 0.054209 | 0.000823 |
| 16 | PHE | -0.08777 | 0.002832 | 0.057713 | 0.001037 |
| 17 | SER | -0.11005 | 0.002526 | 0.078024 | 0.001374 |
| 18 | LEU | -0.05571 | 0.001913 | 0.085995 | 0.000769 |
| 19 | PHE | -0.04797 | 0.002872 | 0.05232 | 0.000987 |
| 20 | ASP | -0.06235 | 0.003133 | -0.91249 | 0.002953 |
| 21 | LYS | -0.07005 | 0.002342 | 0.991401 | 0.002886 |
| 22 | ASP | -0.05116 | 0.001981 | -0.92162 | 0.003011 |
| 23 | GLY | -0.19028 | 0.001965 | 0.203215 | 0.000858 |
| 24 | ASP | -0.06995 | 0.001621 | -0.95153 | 0.002709 |
| 25 | GLY | -0.15163 | 0.002246 | 0.196341 | 0.000873 |
| 26 | THR | -0.07295 | 0.002734 | 0.062284 | 0.001389 |
| 27 | ILE | -0.07781 | 0.002735 | 0.094598 | 0.001014 |
| 28 | THR | -0.12315 | 0.002981 | 0.095029 | 0.001383 |
| 29 | THR | -0.12971 | 0.003172 | 0.09775 | 0.001452 |
| 30 | LYS | -0.07698 | 0.002605 | 0.995746 | 0.003 |
| 31 | GLU | -0.04042 | 0.002488 | -0.8705 | 0.002936 |
| 32 | LEU | -0.10614 | 0.003005 | 0.073787 | 0.00101 |
| 33 | GLY | -0.18567 | 0.002592 | 0.19735 | 0.001139 |
| 34 | THR | -0.11225 | 0.002544 | 0.107306 | 0.001211 |
| 35 | VAL | -0.09447 | 0.00297 | 0.112614 | 0.001128 |
| 36 | MET | -0.07827 | 0.002824 | 0.068951 | 0.000918 |
| 37 | ARG | -0.07953 | 0.002455 | 1.0216 | 0.003287 |
| 38 | SER | -0.10028 | 0.002563 | 0.064614 | 0.001305 |
| 39 | LEU | -0.07642 | 0.002465 | 0.086698 | 0.000744 |
| 40 | GLY | -0.21243 | 0.002152 | 0.20206 | 0.000809 |
| 41 | GLN | -0.03978 | 0.001984 | 0.064189 | 0.000931 |
| 42 | ASN | -0.03522 | 0.00342 | -0.00056 | 0.000877 |
| 43 | PRO | -0.27977 | 0.003024 | 0.296443 | 0.001538 |
| 44 | THR | -0.10634 | 0.002106 | 0.09309 | 0.001078 |
| 45 | GLU | -0.07994 | 0.002264 | -0.87353 | 0.003 |
| 46 | ALA | -0.05092 | 0.002502 | 0.050517 | 0.000643 |
| 47 | GLU | -0.06868 | 0.002725 | -0.87223 | 0.002952 |
| 48 | LEU | -0.07185 | 0.002562 | 0.080496 | 0.000923 |
| 49 | GLN | -0.08393 | 0.002598 | 0.057756 | 0.00111 |
| 50 | ASP | -0.04237 | 0.002541 | -0.95039 | 0.002796 |
| 51 | MET | -0.03567 | 0.00281 | 0.069788 | 0.000856 |
| 52 | ILE | -0.11523 | 0.003108 | 0.125823 | 0.001147 |
| 53 | ASN | -0.05389 | 0.002662 | -0.00132 | 0.000964 |
| 54 | GLU | -0.07441 | 0.002284 | -0.89417 | 0.002756 |
| 55 | VAL | -0.09465 | 0.00283 | 0.129417 | 0.001064 |
| 56 | ASP | -0.06671 | 0.002827 | -0.90632 | 0.002919 |
| 57 | ALA | -0.06006 | 0.002219 | 0.053299 | 0.000613 |
| 58 | ASP | -0.05322 | 0.002114 | -0.9273 | 0.002937 |
| 59 | GLY | -0.19571 | 0.001744 | 0.20393 | 0.000665 |
| 60 | ASN | -0.05077 | 0.001593 | -0.00868 | 0.00071 |
| 61 | GLY | -0.16043 | 0.002459 | 0.199659 | 0.000979 |
| 62 | THR | -0.10097 | 0.003017 | 0.074418 | 0.001381 |
| 63 | ILE | -0.09365 | 0.002759 | 0.102393 | 0.001158 |
| 64 | ASP | -0.02933 | 0.002535 | -0.95051 | 0.002858 |
| 65 | PHE | -0.04717 | 0.003357 | 0.036799 | 0.000868 |
| 66 | PRO | -0.26149 | 0.003033 | 0.285524 | 0.00163 |
| 67 | GLU | -0.07688 | 0.002608 | -0.84611 | 0.002994 |
| 68 | PHE | -0.03735 | 0.002831 | 0.040975 | 0.000917 |
| 69 | LEU | -0.04746 | 0.003307 | 0.077073 | 0.000975 |
| 70 | THR | -0.14809 | 0.003017 | 0.100673 | 0.001388 |
| 71 | MET | -0.05415 | 0.002905 | 0.068694 | 0.00096 |
| 72 | MET | -0.06983 | 0.003086 | 0.066514 | 0.00099 |
| 73 | ALA | -0.03766 | 0.003148 | 0.044645 | 0.001021 |
| 74 | ARG | -0.07731 | 0.003026 | 0.97289 | 0.003094 |
| 75 | LYS | -0.07735 | 0.003001 | 1.00246 | 0.003074 |
| 76 | MET | -0.07777 | 0.003382 | 0.083209 | 0.001238 |
| 77 | LYS | -0.08817 | 0.00301 | 0.994763 | 0.003019 |
| 78 | ASP | -0.03051 | 0.002623 | -0.91802 | 0.002963 |
| 79 | THR | -0.11482 | 0.002455 | 0.10748 | 0.001117 |
| 80 | ASP | -0.04277 | 0.002534 | -0.91343 | 0.003088 |
| 81 | SER | -0.08902 | 0.002807 | 0.072827 | 0.001439 |
| 82 | GLU | -0.08207 | 0.002402 | -0.84015 | 0.002831 |
| 83 | GLU | -0.07916 | 0.002326 | -0.87383 | 0.002932 |
| 84 | GLU | -0.08282 | 0.002649 | -0.88242 | 0.002812 |
| 85 | ILE | -0.08287 | 0.002873 | 0.112958 | 0.000899 |
| 86 | ARG | -0.08752 | 0.002543 | 0.951075 | 0.004047 |
| 87 | GLU | -0.06782 | 0.002596 | -0.84039 | 0.002938 |
| 88 | ALA | -0.0549 | 0.002947 | 0.053755 | 0.000958 |
| 89 | PHE | -0.05904 | 0.002991 | 0.057419 | 0.001 |
| 90 | ARG | -0.0969 | 0.002945 | 0.969427 | 0.003135 |
| 91 | VAL | -0.09636 | 0.002894 | 0.12788 | 0.00093 |
| 92 | PHE | -0.06888 | 0.002594 | 0.057858 | 0.001028 |
| 93 | ASP | -0.06855 | 0.003009 | -0.90209 | 0.002877 |
| 94 | LYS | -0.0647 | 0.002401 | 0.998536 | 0.00311 |
| 95 | ASP | -0.06529 | 0.00196 | -0.946 | 0.002773 |
| 96 | GLY | -0.18284 | 0.001704 | 0.204254 | 0.000708 |
| 97 | ASN | -0.03917 | 0.001776 | -0.00759 | 0.000668 |
| 98 | GLY | -0.16563 | 0.002367 | 0.197566 | 0.001056 |
| 99 | TYR | -0.04931 | 0.002169 | 0.039298 | 0.00266 |
| 100 | ILE | -0.07684 | 0.002101 | 0.106268 | 0.000946 |
| 101 | SER | -0.11629 | 0.002717 | 0.078228 | 0.001351 |
| 102 | ALA | -0.06396 | 0.003264 | 0.056417 | 0.001318 |
| 103 | ALA | -0.02829 | 0.002612 | 0.052841 | 0.00074 |
| 104 | GLU | -0.06937 | 0.002752 | -0.86071 | 0.002822 |
| 105 | LEU | -0.09033 | 0.002841 | 0.071837 | 0.001095 |
| 106 | ARG | -0.0672 | 0.002624 | 0.965661 | 0.00321 |
| 107 | HIS | -0.04575 | 0.002632 | 0.05153 | 0.001015 |
| 108 | VAL | -0.09738 | 0.003149 | 0.105497 | 0.001073 |
| 109 | MET | -0.07639 | 0.003192 | 0.064633 | 0.000907 |
| 110 | THR | -0.11226 | 0.002788 | 0.096856 | 0.001238 |
| 111 | ASN | -0.03628 | 0.002376 | -0.00094 | 0.00079 |
| 112 | LEU | -0.08426 | 0.002447 | 0.08578 | 0.000795 |
| 113 | GLY | -0.21009 | 0.002326 | 0.201367 | 0.000985 |
| 114 | GLU | -0.07717 | 0.002251 | -0.86215 | 0.00278 |
| 115 | LYS | -0.07376 | 0.002002 | 0.972794 | 0.002999 |
| 116 | LEU | -0.0644 | 0.002144 | 0.073582 | 0.000736 |
| 117 | THR | -0.12002 | 0.002403 | 0.096525 | 0.001114 |
| 118 | ASP | -0.02637 | 0.002096 | -0.916 | 0.003063 |
| 119 | GLU | -0.08527 | 0.001839 | -0.91836 | 0.002549 |
| 120 | GLU | -0.06015 | 0.002145 | -0.87463 | 0.002868 |
| 121 | VAL | -0.11231 | 0.002487 | 0.111869 | 0.000883 |
| 122 | ASP | -0.036 | 0.002385 | -0.91103 | 0.002947 |
| 123 | GLU | -0.07115 | 0.00228 | -0.86328 | 0.002889 |
| 124 | MET | -0.05806 | 0.002551 | 0.065938 | 0.000797 |
| 125 | ILE | -0.124 | 0.002977 | 0.11228 | 0.001106 |
| 126 | ARG | -0.07888 | 0.002158 | 0.944073 | 0.003082 |
| 127 | GLU | -0.0777 | 0.00181 | -0.89244 | 0.002884 |
| 128 | ALA | -0.04984 | 0.002042 | 0.049453 | 0.000795 |
| 129 | ASP | -0.00949 | 0.002525 | -0.94425 | 0.002854 |
| 130 | ILE | -0.11523 | 0.002503 | 0.107585 | 0.000856 |
| 131 | ASP | -0.05219 | 0.002277 | -0.94319 | 0.002906 |
| 132 | GLY | -0.20554 | 0.002018 | 0.201153 | 0.000649 |
| 133 | ASP | -0.03854 | 0.001589 | -0.95445 | 0.002717 |
| 134 | GLY | -0.18784 | 0.002231 | 0.194059 | 0.000852 |
| 135 | GLN | -0.05763 | 0.002365 | 0.040669 | 0.001123 |
| 136 | VAL | -0.06466 | 0.002149 | 0.104138 | 0.000746 |
| 137 | ASN | -0.01815 | 0.002084 | -0.00487 | 0.000926 |
| 138 | TYR | -0.06706 | 0.002975 | 0.038031 | 0.001462 |
| 139 | GLU | -0.08 | 0.002627 | -0.89445 | 0.002818 |
| 140 | GLU | -0.06093 | 0.002224 | -0.88082 | 0.002768 |
| 141 | PHE | -0.05471 | 0.002631 | 0.053913 | 0.000861 |
| 142 | VAL | -0.12851 | 0.003008 | 0.11804 | 0.001045 |
| 143 | GLN | -0.06459 | 0.002713 | 0.062301 | 0.001063 |
| 144 | MET | -0.0544 | 0.002693 | 0.061409 | 0.000837 |
| 145 | MET | -0.04358 | 0.002714 | 0.067628 | 0.000882 |
| 146 | THR | -0.13363 | 0.003088 | 0.083885 | 0.001356 |
| 147 | ALA | -0.02698 | 0.002772 | 0.033397 | 0.001001 |
| 148 | LYS | -0.06129 | 0.001917 | 0.993553 | 0.003026 |

Table S2. Charge distribution on a coarse-grained C_α_-side chain model of holoCaM. The unit of charge is 1.6*10^-19^ C.

| Residue  Index | Residue Name | Charge (C_α_) | Error (C_α_) | Charge  (side chain) | Error  (side chain) |
| --- | --- | --- | --- | --- | --- |
| 1 | ALA | -0.08329 | 0.001822 | 0.042999 | 0.000628 |
| 2 | ASP | -0.01905 | 0.001941 | -0.93543 | 0.001674 |
| 3 | GLN | -0.04978 | 0.002241 | 0.049023 | 0.001071 |
| 4 | LEU | -0.07136 | 0.00222 | 0.076875 | 0.000851 |
| 5 | THR | -0.11342 | 0.002618 | 0.086196 | 0.001306 |
| 6 | GLU | -0.07723 | 0.002236 | -0.87116 | 0.001918 |
| 7 | GLU | -0.08581 | 0.002398 | -0.89148 | 0.001857 |
| 8 | GLN | -0.05752 | 0.002789 | 0.05923 | 0.001067 |
| 9 | ILE | -0.11565 | 0.00259 | 0.113847 | 0.000892 |
| 10 | ALA | -0.05849 | 0.002291 | 0.051815 | 0.000652 |
| 11 | GLU | -0.06742 | 0.002506 | -0.87495 | 0.001799 |
| 12 | PHE | -0.0512 | 0.002568 | 0.050465 | 0.000895 |
| 13 | LYS | -0.08022 | 0.002357 | 0.995625 | 0.001479 |
| 14 | GLU | -0.07277 | 0.002337 | -0.88545 | 0.001715 |
| 15 | ALA | -0.03742 | 0.002363 | 0.049033 | 0.000929 |
| 16 | PHE | -0.07165 | 0.002595 | 0.053093 | 0.000889 |
| 17 | SER | -0.10152 | 0.00249 | 0.077844 | 0.001222 |
| 18 | LEU | -0.05348 | 0.002303 | 0.084082 | 0.00084 |
| 19 | PHE | -0.03522 | 0.003004 | 0.04721 | 0.001051 |
| 20 | ASP | -0.06143 | 0.003098 | -0.8993 | 0.004403 |
| 21 | LYS | -0.04839 | 0.002552 | 1.01676 | 0.001718 |
| 22 | ASP | -0.05995 | 0.002242 | -0.94083 | 0.003278 |
| 23 | GLY | -0.18179 | 0.00233 | 0.202989 | 0.00093 |
| 24 | ASP | -0.06328 | 0.002136 | -0.92702 | 0.004757 |
| 25 | GLY | -0.15876 | 0.002165 | 0.202635 | 0.000841 |
| 26 | THR | -0.12835 | 0.003482 | 0.10075 | 0.002262 |
| 27 | ILE | -0.09418 | 0.003117 | 0.117609 | 0.001091 |
| 28 | THR | -0.10275 | 0.003262 | 0.103029 | 0.001333 |
| 29 | THR | -0.13243 | 0.003284 | 0.09773 | 0.001503 |
| 30 | LYS | -0.06356 | 0.002907 | 1.00005 | 0.001563 |
| 31 | GLU | -0.06035 | 0.00278 | -0.83782 | 0.001983 |
| 32 | LEU | -0.0697 | 0.002911 | 0.075841 | 0.000952 |
| 33 | GLY | -0.20876 | 0.002548 | 0.196785 | 0.000836 |
| 34 | THR | -0.11839 | 0.002375 | 0.100872 | 0.001065 |
| 35 | VAL | -0.08591 | 0.002489 | 0.114564 | 0.00077 |
| 36 | MET | -0.05864 | 0.002416 | 0.061806 | 0.000764 |
| 37 | ARG | -0.07187 | 0.002447 | 1.01464 | 0.001799 |
| 38 | SER | -0.07995 | 0.002448 | 0.065793 | 0.001448 |
| 39 | LEU | -0.07783 | 0.002532 | 0.082115 | 0.000921 |
| 40 | GLY | -0.21212 | 0.002757 | 0.19624 | 0.000768 |
| 41 | GLN | -0.05608 | 0.002265 | 0.062049 | 0.000922 |
| 42 | ASN | -0.0547 | 0.003024 | 0.000682 | 0.001108 |
| 43 | PRO | -0.26095 | 0.003156 | 0.295906 | 0.0014 |
| 44 | THR | -0.09927 | 0.002559 | 0.092661 | 0.001101 |
| 45 | GLU | -0.08411 | 0.002563 | -0.87678 | 0.00208 |
| 46 | ALA | -0.04937 | 0.002188 | 0.049155 | 0.000664 |
| 47 | GLU | -0.05961 | 0.002315 | -0.87572 | 0.001814 |
| 48 | LEU | -0.06352 | 0.002653 | 0.079209 | 0.001084 |
| 49 | GLN | -0.09299 | 0.002566 | 0.050768 | 0.001018 |
| 50 | ASP | -0.02056 | 0.002121 | -0.95506 | 0.001019 |
| 51 | MET | -0.05071 | 0.002461 | 0.069042 | 0.000869 |
| 52 | ILE | -0.1007 | 0.003094 | 0.111263 | 0.000928 |
| 53 | ASN | -0.05505 | 0.002559 | 9.64E-05 | 0.001075 |
| 54 | GLU | -0.06443 | 0.002 | -0.88243 | 0.002099 |
| 55 | VAL | -0.06901 | 0.002569 | 0.123052 | 0.000819 |
| 56 | ASP | -0.04532 | 0.003805 | -0.86573 | 0.006724 |
| 57 | ALA | 0.002813 | 0.004016 | 0.073909 | 0.000926 |
| 58 | ASP | -0.06965 | 0.001658 | -0.95617 | 0.001186 |
| 59 | GLY | -0.17402 | 0.001423 | 0.209407 | 0.000675 |
| 60 | ASN | -0.04878 | 0.001991 | 0.021289 | 0.003085 |
| 61 | GLY | -0.15612 | 0.002248 | 0.206191 | 0.000751 |
| 62 | THR | -0.12642 | 0.003262 | 0.095492 | 0.001652 |
| 63 | ILE | -0.12169 | 0.003258 | 0.117642 | 0.001976 |
| 64 | ASP | -0.03425 | 0.002649 | -0.93648 | 0.001663 |
| 65 | PHE | -0.04205 | 0.003656 | 0.044643 | 0.000893 |
| 66 | PRO | -0.27092 | 0.003235 | 0.292146 | 0.001485 |
| 67 | GLU | -0.07051 | 0.002161 | -0.87807 | 0.004404 |
| 68 | PHE | -0.05307 | 0.002801 | 0.040641 | 0.000951 |
| 69 | LEU | -0.0448 | 0.002697 | 0.075945 | 0.001052 |
| 70 | THR | -0.12755 | 0.002403 | 0.095061 | 0.001208 |
| 71 | MET | -0.04968 | 0.00214 | 0.054077 | 0.001189 |
| 72 | MET | -0.06094 | 0.002523 | 0.061632 | 0.000897 |
| 73 | ALA | -0.03885 | 0.002601 | 0.044031 | 0.000857 |
| 74 | ARG | -0.07033 | 0.002494 | 0.986646 | 0.002073 |
| 75 | LYS | -0.06263 | 0.002526 | 0.98591 | 0.001721 |
| 76 | MET | -0.05882 | 0.002635 | 0.064228 | 0.000933 |
| 77 | LYS | -0.08604 | 0.002608 | 0.991131 | 0.001562 |
| 78 | ASP | -0.0224 | 0.002577 | -0.91311 | 0.001645 |
| 79 | THR | -0.12237 | 0.002535 | 0.094993 | 0.001202 |
| 80 | ASP | -0.01423 | 0.002227 | -0.90474 | 0.001562 |
| 81 | SER | -0.09207 | 0.002207 | 0.068059 | 0.001141 |
| 82 | GLU | -0.07473 | 0.002097 | -0.86736 | 0.002152 |
| 83 | GLU | -0.06949 | 0.001899 | -0.88268 | 0.001839 |
| 84 | GLU | -0.06689 | 0.002789 | -0.86063 | 0.001692 |
| 85 | ILE | -0.11617 | 0.002767 | 0.118179 | 0.001015 |
| 86 | ARG | -0.08337 | 0.002197 | 0.958494 | 0.001986 |
| 87 | GLU | -0.06123 | 0.002653 | -0.85992 | 0.001906 |
| 88 | ALA | -0.05409 | 0.002555 | 0.052382 | 0.000938 |
| 89 | PHE | -0.03132 | 0.002638 | 0.054217 | 0.000905 |
| 90 | ARG | -0.08107 | 0.002728 | 1.00853 | 0.003213 |
| 91 | VAL | -0.11678 | 0.003248 | 0.121488 | 0.001369 |
| 92 | PHE | -0.03381 | 0.00299 | 0.061281 | 0.001186 |
| 93 | ASP | -0.06126 | 0.004537 | -0.80186 | 0.010099 |
| 94 | LYS | -0.00357 | 0.005024 | 1.03895 | 0.002188 |
| 95 | ASP | -0.06736 | 0.002691 | -0.92346 | 0.004031 |
| 96 | GLY | -0.17784 | 0.002395 | 0.20319 | 0.000927 |
| 97 | ASN | -0.05213 | 0.002191 | 0.019857 | 0.003282 |
| 98 | GLY | -0.16585 | 0.00174 | 0.202501 | 0.000707 |
| 99 | TYR | -0.06014 | 0.002459 | 0.053078 | 0.001145 |
| 100 | ILE | -0.10469 | 0.002667 | 0.109076 | 0.002767 |
| 101 | SER | -0.09558 | 0.002718 | 0.079605 | 0.001311 |
| 102 | ALA | -0.0485 | 0.002699 | 0.052874 | 0.000907 |
| 103 | ALA | -0.02854 | 0.002364 | 0.056422 | 0.000709 |
| 104 | GLU | -0.06991 | 0.002751 | -0.83625 | 0.005166 |
| 105 | LEU | -0.0834 | 0.002789 | 0.078237 | 0.001089 |
| 106 | ARG | -0.06001 | 0.002541 | 0.983237 | 0.002014 |
| 107 | HIS | -0.05943 | 0.002672 | 0.052804 | 0.001071 |
| 108 | VAL | -0.11215 | 0.002653 | 0.106661 | 0.000919 |
| 109 | MET | -0.06374 | 0.002851 | 0.069078 | 0.000893 |
| 110 | THR | -0.1233 | 0.00269 | 0.111593 | 0.001147 |
| 111 | ASN | -0.02456 | 0.002248 | 0.003252 | 0.000955 |
| 112 | LEU | -0.08074 | 0.002707 | 0.081453 | 0.000851 |
| 113 | GLY | -0.21223 | 0.002648 | 0.200144 | 0.000722 |
| 114 | GLU | -0.08415 | 0.002907 | -0.85217 | 0.001746 |
| 115 | LYS | -0.07031 | 0.002636 | 0.980574 | 0.001881 |
| 116 | LEU | -0.04618 | 0.002554 | 0.069973 | 0.000838 |
| 117 | THR | -0.1242 | 0.002673 | 0.096744 | 0.001237 |
| 118 | ASP | -0.02475 | 0.00233 | -0.90793 | 0.001806 |
| 119 | GLU | -0.07787 | 0.002208 | -0.91612 | 0.000969 |
| 120 | GLU | -0.07634 | 0.002682 | -0.86302 | 0.001965 |
| 121 | VAL | -0.11239 | 0.00314 | 0.113562 | 0.000985 |
| 122 | ASP | -0.05359 | 0.002847 | -0.90835 | 0.001673 |
| 123 | GLU | -0.08131 | 0.002624 | -0.87383 | 0.001953 |
| 124 | MET | -0.04502 | 0.002513 | 0.065233 | 0.000848 |
| 125 | ILE | -0.1193 | 0.002956 | 0.105997 | 0.001042 |
| 126 | ARG | -0.0793 | 0.002326 | 0.961834 | 0.002669 |
| 127 | GLU | -0.06434 | 0.002097 | -0.87508 | 0.002152 |
| 128 | ALA | -0.02739 | 0.002529 | 0.04199 | 0.000928 |
| 129 | ASP | -0.03922 | 0.002849 | -0.87128 | 0.006104 |
| 130 | ILE | -0.06841 | 0.002669 | 0.128339 | 0.000883 |
| 131 | ASP | -0.01789 | 0.003463 | -0.89015 | 0.008149 |
| 132 | GLY | -0.13353 | 0.005276 | 0.222293 | 0.001679 |
| 133 | ASP | -0.07503 | 0.003435 | -0.88114 | 0.008498 |
| 134 | GLY | -0.17466 | 0.003862 | 0.208725 | 0.001025 |
| 135 | GLN | -0.06982 | 0.002377 | 0.051196 | 0.002232 |
| 136 | VAL | -0.09037 | 0.002307 | 0.104689 | 0.000979 |
| 137 | ASN | -0.01737 | 0.002209 | -0.00929 | 0.001102 |
| 138 | TYR | -0.06168 | 0.002375 | 0.044186 | 0.001255 |
| 139 | GLU | -0.06682 | 0.002636 | -0.89441 | 0.001855 |
| 140 | GLU | -0.06956 | 0.002707 | -0.85163 | 0.004399 |
| 141 | PHE | -0.05339 | 0.00299 | 0.045793 | 0.001038 |
| 142 | VAL | -0.10521 | 0.002771 | 0.124559 | 0.001205 |
| 143 | GLN | -0.0565 | 0.002729 | 0.057119 | 0.001152 |
| 144 | MET | -0.07063 | 0.002768 | 0.062833 | 0.00107 |
| 145 | MET | -0.06187 | 0.002678 | 0.065834 | 0.001033 |
| 146 | THR | -0.09524 | 0.003022 | 0.09151 | 0.001554 |
| 147 | ALA | -0.04167 | 0.002616 | 0.035988 | 0.001296 |
| 148 | LYS | -0.07747 | 0.001894 | 0.988067 | 0.001859 |

Table S3. Contact pair index (CPI) of apoCaM. “b” denotes contacts between side chain beads and “h” denotes contacts between C_α_ beads.

| Contact Pair Index (CPI) | Type | Reside  Index | Residue Index | Contact Pair  Index (CPI) | Type | Residue  Index | Residue Index |
| --- | --- | --- | --- | --- | --- | --- | --- |
| 1 | b | 1 | 3 | 206 | b | 94 | 104 |
| 2 | b | 1 | 147 | 207 | b | 95 | 97 |
| 3 | b | 2 | 76 | 208 | b | 97 | 99 |
| 4 | b | 2 | 77 | 209 | b | 99 | 135 |
| 5 | b | 2 | 80 | 210 | b | 99 | 136 |
| 6 | b | 3 | 147 | 211 | b | 99 | 137 |
| 7 | b | 4 | 8 | 212 | b | 100 | 104 |
| 8 | b | 4 | 9 | 213 | b | 100 | 105 |
| 9 | b | 4 | 12 | 214 | b | 100 | 108 |
| 10 | b | 4 | 69 | 215 | b | 100 | 138 |
| 11 | b | 4 | 72 | 216 | b | 101 | 103 |
| 12 | b | 4 | 73 | 217 | b | 101 | 104 |
| 13 | b | 4 | 76 | 218 | b | 101 | 135 |
| 14 | b | 5 | 7 | 219 | b | 102 | 106 |
| 15 | b | 5 | 8 | 220 | b | 102 | 130 |
| 16 | b | 6 | 9 | 221 | b | 104 | 107 |
| 17 | b | 7 | 11 | 222 | b | 105 | 109 |
| 18 | b | 8 | 11 | 223 | b | 105 | 125 |
| 19 | b | 8 | 12 | 224 | b | 105 | 130 |
| 20 | b | 8 | 76 | 225 | b | 105 | 136 |
| 21 | b | 8 | 81 | 226 | b | 105 | 138 |
| 22 | b | 9 | 13 | 227 | b | 105 | 141 |
| 23 | b | 9 | 65 | 228 | b | 106 | 110 |
| 24 | b | 9 | 69 | 229 | b | 106 | 118 |
| 25 | b | 10 | 14 | 230 | b | 106 | 121 |
| 26 | b | 11 | 39 | 231 | b | 106 | 122 |
| 27 | b | 12 | 39 | 232 | b | 106 | 125 |
| 28 | b | 12 | 65 | 233 | b | 107 | 110 |
| 29 | b | 12 | 68 | 234 | b | 107 | 111 |
| 30 | b | 12 | 69 | 235 | b | 108 | 112 |
| 31 | b | 12 | 72 | 236 | b | 109 | 116 |
| 32 | b | 12 | 76 | 237 | b | 109 | 121 |
| 33 | b | 13 | 16 | 238 | b | 109 | 124 |
| 34 | b | 13 | 17 | 239 | b | 109 | 141 |
| 35 | b | 13 | 65 | 240 | b | 109 | 145 |
| 36 | b | 14 | 18 | 241 | b | 110 | 116 |
| 37 | b | 15 | 18 | 242 | b | 110 | 118 |
| 38 | b | 15 | 35 | 243 | b | 110 | 121 |
| 39 | b | 15 | 38 | 244 | b | 112 | 114 |
| 40 | b | 15 | 39 | 245 | b | 112 | 145 |
| 41 | b | 16 | 20 | 246 | b | 114 | 116 |
| 42 | b | 16 | 27 | 247 | b | 114 | 145 |
| 43 | b | 16 | 35 | 248 | b | 116 | 120 |
| 44 | b | 16 | 63 | 249 | b | 116 | 121 |
| 45 | b | 16 | 65 | 250 | b | 116 | 124 |
| 46 | b | 16 | 68 | 251 | b | 116 | 145 |
| 47 | b | 17 | 20 | 252 | b | 117 | 119 |
| 48 | b | 18 | 38 | 253 | b | 117 | 120 |
| 49 | b | 19 | 21 | 254 | b | 118 | 121 |
| 50 | b | 19 | 27 | 255 | b | 118 | 122 |
| 51 | b | 19 | 31 | 256 | b | 119 | 122 |
| 52 | b | 19 | 34 | 257 | b | 120 | 123 |
| 53 | b | 19 | 35 | 258 | b | 120 | 124 |
| 54 | b | 19 | 38 | 259 | b | 121 | 124 |
| 55 | b | 20 | 24 | 260 | b | 121 | 125 |
| 56 | b | 20 | 27 | 261 | b | 121 | 141 |
| 57 | b | 21 | 31 | 262 | b | 122 | 125 |
| 58 | b | 22 | 24 | 263 | b | 122 | 126 |
| 59 | b | 22 | 26 | 264 | b | 123 | 126 |
| 60 | b | 22 | 28 | 265 | b | 123 | 148 |
| 61 | b | 24 | 26 | 266 | b | 124 | 141 |
| 62 | b | 26 | 62 | 267 | b | 124 | 144 |
| 63 | b | 26 | 64 | 268 | b | 124 | 145 |
| 64 | b | 27 | 31 | 269 | b | 124 | 148 |
| 65 | b | 27 | 32 | 270 | b | 125 | 130 |
| 66 | b | 27 | 35 | 271 | b | 125 | 141 |
| 67 | b | 27 | 63 | 272 | b | 127 | 144 |
| 68 | b | 27 | 68 | 273 | b | 128 | 130 |
| 69 | b | 28 | 30 | 274 | b | 128 | 136 |
| 70 | b | 28 | 31 | 275 | b | 128 | 140 |
| 71 | b | 28 | 62 | 276 | b | 128 | 141 |
| 72 | b | 29 | 52 | 277 | b | 128 | 144 |
| 73 | b | 29 | 55 | 278 | b | 130 | 136 |
| 74 | b | 29 | 56 | 279 | b | 130 | 141 |
| 75 | b | 29 | 63 | 280 | b | 133 | 135 |
| 76 | b | 31 | 34 | 281 | b | 133 | 136 |
| 77 | b | 31 | 35 | 282 | b | 136 | 140 |
| 78 | b | 32 | 35 | 283 | b | 136 | 141 |
| 79 | b | 32 | 36 | 284 | b | 137 | 139 |
| 80 | b | 32 | 48 | 285 | b | 137 | 140 |
| 81 | b | 32 | 51 | 286 | b | 138 | 142 |
| 82 | b | 32 | 52 | 287 | b | 139 | 142 |
| 83 | b | 32 | 63 | 288 | b | 139 | 143 |
| 84 | b | 32 | 68 | 289 | b | 140 | 144 |
| 85 | b | 32 | 71 | 290 | b | 141 | 144 |
| 86 | b | 34 | 37 | 291 | b | 144 | 148 |
| 87 | b | 34 | 38 | 292 | h | 1 | 81 |
| 88 | b | 35 | 39 | 293 | h | 1 | 82 |
| 89 | b | 35 | 68 | 294 | h | 2 | 80 |
| 90 | b | 36 | 39 | 295 | h | 2 | 81 |
| 91 | b | 36 | 41 | 296 | h | 4 | 8 |
| 92 | b | 36 | 43 | 297 | h | 5 | 9 |
| 93 | b | 36 | 48 | 298 | h | 5 | 10 |
| 94 | b | 36 | 51 | 299 | h | 6 | 10 |
| 95 | b | 36 | 68 | 300 | h | 7 | 11 |
| 96 | b | 36 | 71 | 301 | h | 7 | 12 |
| 97 | b | 36 | 72 | 302 | h | 8 | 12 |
| 98 | b | 36 | 75 | 303 | h | 8 | 13 |
| 99 | b | 37 | 42 | 304 | h | 9 | 13 |
| 100 | b | 37 | 43 | 305 | h | 10 | 14 |
| 101 | b | 37 | 48 | 306 | h | 10 | 15 |
| 102 | b | 39 | 41 | 307 | h | 11 | 15 |
| 103 | b | 39 | 68 | 308 | h | 11 | 16 |
| 104 | b | 39 | 72 | 309 | h | 12 | 16 |
| 105 | b | 41 | 43 | 310 | h | 12 | 17 |
| 106 | b | 41 | 72 | 311 | h | 13 | 17 |
| 107 | b | 41 | 75 | 312 | h | 14 | 18 |
| 108 | b | 41 | 79 | 313 | h | 15 | 19 |
| 109 | b | 42 | 44 | 314 | h | 19 | 28 |
| 110 | b | 43 | 47 | 315 | h | 20 | 25 |
| 111 | b | 43 | 48 | 316 | h | 21 | 27 |
| 112 | b | 43 | 51 | 317 | h | 22 | 26 |
| 113 | b | 43 | 75 | 318 | h | 25 | 65 |
| 114 | b | 44 | 46 | 319 | h | 26 | 64 |
| 115 | b | 44 | 47 | 320 | h | 27 | 63 |
| 116 | b | 45 | 48 | 321 | h | 28 | 32 |
| 117 | b | 45 | 49 | 322 | h | 29 | 61 |
| 118 | b | 46 | 50 | 323 | h | 29 | 33 |
| 119 | b | 47 | 51 | 324 | h | 30 | 61 |
| 120 | b | 47 | 75 | 325 | h | 30 | 34 |
| 121 | b | 48 | 51 | 326 | h | 31 | 35 |
| 122 | b | 48 | 52 | 327 | h | 32 | 36 |
| 123 | b | 49 | 52 | 328 | h | 32 | 37 |
| 124 | b | 51 | 55 | 329 | h | 33 | 37 |
| 125 | b | 51 | 71 | 330 | h | 33 | 38 |
| 126 | b | 51 | 75 | 331 | h | 34 | 38 |
| 127 | b | 52 | 55 | 332 | h | 35 | 39 |
| 128 | b | 52 | 56 | 333 | h | 36 | 41 |
| 129 | b | 52 | 63 | 334 | h | 44 | 48 |
| 130 | b | 54 | 74 | 335 | h | 44 | 49 |
| 131 | b | 55 | 63 | 336 | h | 45 | 49 |
| 132 | b | 55 | 67 | 337 | h | 45 | 50 |
| 133 | b | 55 | 70 | 338 | h | 46 | 50 |
| 134 | b | 55 | 71 | 339 | h | 46 | 51 |
| 135 | b | 58 | 60 | 340 | h | 47 | 51 |
| 136 | b | 60 | 62 | 341 | h | 48 | 52 |
| 137 | b | 63 | 67 | 342 | h | 49 | 53 |
| 138 | b | 63 | 68 | 343 | h | 50 | 54 |
| 139 | b | 63 | 71 | 344 | h | 51 | 55 |
| 140 | b | 64 | 66 | 345 | h | 51 | 56 |
| 141 | b | 64 | 67 | 346 | h | 52 | 56 |
| 142 | b | 65 | 68 | 347 | h | 58 | 62 |
| 143 | b | 65 | 69 | 348 | h | 64 | 68 |
| 144 | b | 67 | 70 | 349 | h | 65 | 69 |
| 145 | b | 68 | 71 | 350 | h | 65 | 70 |
| 146 | b | 68 | 72 | 351 | h | 66 | 70 |
| 147 | b | 70 | 73 | 352 | h | 67 | 71 |
| 148 | b | 70 | 74 | 353 | h | 68 | 72 |
| 149 | b | 72 | 75 | 354 | h | 69 | 73 |
| 150 | b | 72 | 76 | 355 | h | 70 | 74 |
| 151 | b | 74 | 77 | 356 | h | 71 | 75 |
| 152 | b | 75 | 79 | 357 | h | 72 | 76 |
| 153 | b | 76 | 79 | 358 | h | 74 | 78 |
| 154 | b | 78 | 83 | 359 | h | 75 | 79 |
| 155 | b | 79 | 84 | 360 | h | 76 | 81 |
| 156 | b | 80 | 82 | 361 | h | 80 | 84 |
| 157 | b | 80 | 83 | 362 | h | 81 | 85 |
| 158 | b | 80 | 146 | 363 | h | 82 | 86 |
| 159 | b | 81 | 84 | 364 | h | 82 | 87 |
| 160 | b | 81 | 85 | 365 | h | 83 | 87 |
| 161 | b | 81 | 146 | 366 | h | 84 | 88 |
| 162 | b | 82 | 86 | 367 | h | 85 | 89 |
| 163 | b | 82 | 142 | 368 | h | 85 | 90 |
| 164 | b | 82 | 143 | 369 | h | 86 | 90 |
| 165 | b | 82 | 146 | 370 | h | 87 | 91 |
| 166 | b | 83 | 86 | 371 | h | 88 | 92 |
| 167 | b | 83 | 87 | 372 | h | 89 | 93 |
| 168 | b | 84 | 87 | 373 | h | 95 | 99 |
| 169 | b | 84 | 112 | 374 | h | 98 | 138 |
| 170 | b | 85 | 89 | 375 | h | 99 | 137 |
| 171 | b | 85 | 108 | 376 | h | 99 | 138 |
| 172 | b | 85 | 112 | 377 | h | 100 | 136 |
| 173 | b | 85 | 114 | 378 | h | 101 | 105 |
| 174 | b | 85 | 142 | 379 | h | 101 | 135 |
| 175 | b | 85 | 145 | 380 | h | 102 | 134 |
| 176 | b | 85 | 146 | 381 | h | 102 | 106 |
| 177 | b | 86 | 90 | 382 | h | 103 | 107 |
| 178 | b | 86 | 138 | 383 | h | 104 | 108 |
| 179 | b | 86 | 139 | 384 | h | 105 | 109 |
| 180 | b | 86 | 142 | 385 | h | 106 | 110 |
| 181 | b | 87 | 90 | 386 | h | 107 | 111 |
| 182 | b | 88 | 92 | 387 | h | 107 | 112 |
| 183 | b | 88 | 108 | 388 | h | 108 | 112 |
| 184 | b | 88 | 112 | 389 | h | 109 | 114 |
| 185 | b | 89 | 100 | 390 | h | 109 | 113 |
| 186 | b | 89 | 105 | 391 | h | 117 | 121 |
| 187 | b | 89 | 108 | 392 | h | 118 | 122 |
| 188 | b | 89 | 109 | 393 | h | 119 | 123 |
| 189 | b | 89 | 138 | 394 | h | 120 | 124 |
| 190 | b | 89 | 141 | 395 | h | 121 | 125 |
| 191 | b | 89 | 142 | 396 | h | 122 | 126 |
| 192 | b | 89 | 145 | 397 | h | 123 | 127 |
| 193 | b | 90 | 93 | 398 | h | 124 | 128 |
| 194 | b | 90 | 138 | 399 | h | 125 | 130 |
| 195 | b | 91 | 94 | 400 | h | 129 | 133 |
| 196 | b | 92 | 94 | 401 | h | 137 | 141 |
| 197 | b | 92 | 100 | 402 | h | 138 | 142 |
| 198 | b | 92 | 104 | 403 | h | 138 | 143 |
| 199 | b | 92 | 107 | 404 | h | 139 | 143 |
| 200 | b | 92 | 108 | 405 | h | 139 | 144 |
| 201 | b | 92 | 111 | 406 | h | 140 | 144 |
| 202 | b | 93 | 95 | 407 | h | 141 | 145 |
| 203 | b | 93 | 97 | 408 | h | 142 | 146 |
| 204 | b | 93 | 100 | 409 | h | 143 | 147 |
| 205 | b | 93 | 138 | 410 | h | 144 | 148 |

Table S4. Contact pair index (CPI) of holoCaM. “b” denotes contacts between side chain beads and “h” denotes contacts between C_α_ beads.

| Contact Pair Index (CPI) | Type | Reside  Index | Residue Index | Contact Pair  Index (CPI) | Type | Residue  Index | Residue  Index |
| --- | --- | --- | --- | --- | --- | --- | --- |
| 1 | h | 4 | 8 | 190 | b | 29 | 48 |
| 2 | h | 4 | 73 | 191 | b | 29 | 49 |
| 3 | h | 5 | 9 | 192 | b | 29 | 52 |
| 4 | h | 5 | 10 | 193 | b | 29 | 53 |
| 5 | h | 6 | 10 | 194 | b | 30 | 34 |
| 6 | h | 6 | 11 | 195 | b | 30 | 37 |
| 7 | h | 7 | 11 | 196 | b | 32 | 36 |
| 8 | h | 8 | 12 | 197 | b | 32 | 48 |
| 9 | h | 9 | 13 | 198 | b | 32 | 51 |
| 10 | h | 9 | 14 | 199 | b | 32 | 52 |
| 11 | h | 10 | 14 | 200 | b | 32 | 63 |
| 12 | h | 11 | 15 | 201 | b | 34 | 37 |
| 13 | h | 12 | 16 | 202 | b | 34 | 38 |
| 14 | h | 13 | 17 | 203 | b | 35 | 39 |
| 15 | h | 14 | 18 | 204 | b | 35 | 112 |
| 16 | h | 15 | 19 | 205 | b | 36 | 39 |
| 17 | h | 16 | 20 | 206 | b | 36 | 41 |
| 18 | h | 19 | 28 | 207 | b | 36 | 43 |
| 19 | h | 21 | 27 | 208 | b | 36 | 48 |
| 20 | h | 22 | 26 | 209 | b | 36 | 51 |
| 21 | h | 23 | 27 | 210 | b | 37 | 42 |
| 22 | h | 25 | 65 | 211 | b | 37 | 43 |
| 23 | h | 26 | 64 | 212 | b | 37 | 48 |
| 24 | h | 26 | 65 | 213 | b | 38 | 111 |
| 25 | h | 27 | 63 | 214 | b | 38 | 112 |
| 26 | h | 28 | 32 | 215 | b | 39 | 41 |
| 27 | h | 28 | 33 | 216 | b | 39 | 91 |
| 28 | h | 29 | 61 | 217 | b | 39 | 92 |
| 29 | h | 29 | 33 | 218 | b | 39 | 108 |
| 30 | h | 30 | 34 | 219 | b | 39 | 111 |
| 31 | h | 31 | 35 | 220 | b | 39 | 112 |
| 32 | h | 32 | 36 | 221 | b | 41 | 43 |
| 33 | h | 32 | 37 | 222 | b | 41 | 91 |
| 34 | h | 33 | 37 | 223 | b | 43 | 47 |
| 35 | h | 33 | 38 | 224 | b | 43 | 48 |
| 36 | h | 34 | 38 | 225 | b | 43 | 51 |
| 37 | h | 35 | 39 | 226 | b | 44 | 46 |
| 38 | h | 36 | 41 | 227 | b | 44 | 47 |
| 39 | h | 44 | 48 | 228 | b | 45 | 48 |
| 40 | h | 44 | 49 | 229 | b | 45 | 49 |
| 41 | h | 45 | 49 | 230 | b | 46 | 50 |
| 42 | h | 45 | 50 | 231 | b | 47 | 51 |
| 43 | h | 46 | 50 | 232 | b | 49 | 53 |
| 44 | h | 47 | 51 | 233 | b | 50 | 53 |
| 45 | h | 48 | 52 | 234 | b | 50 | 54 |
| 46 | h | 48 | 53 | 235 | b | 51 | 54 |
| 47 | h | 49 | 53 | 236 | b | 52 | 56 |
| 48 | h | 49 | 54 | 237 | b | 52 | 63 |
| 49 | h | 50 | 54 | 238 | b | 53 | 56 |
| 50 | h | 51 | 55 | 239 | b | 55 | 63 |
| 51 | h | 52 | 56 | 240 | b | 55 | 71 |
| 52 | h | 56 | 63 | 241 | b | 56 | 60 |
| 53 | h | 57 | 63 | 242 | b | 57 | 67 |
| 54 | h | 58 | 62 | 243 | b | 58 | 60 |
| 55 | h | 64 | 68 | 244 | b | 60 | 62 |
| 56 | h | 65 | 69 | 245 | b | 60 | 64 |
| 57 | h | 65 | 70 | 246 | b | 60 | 67 |
| 58 | h | 66 | 70 | 247 | b | 63 | 67 |
| 59 | h | 67 | 71 | 248 | b | 63 | 68 |
| 60 | h | 68 | 72 | 249 | b | 63 | 71 |
| 61 | h | 68 | 73 | 250 | b | 64 | 66 |
| 62 | h | 69 | 73 | 251 | b | 65 | 68 |
| 63 | h | 69 | 74 | 252 | b | 65 | 69 |
| 64 | h | 70 | 74 | 253 | b | 68 | 71 |
| 65 | h | 71 | 75 | 254 | b | 68 | 72 |
| 66 | h | 72 | 76 | 255 | b | 69 | 73 |
| 67 | h | 73 | 77 | 256 | b | 70 | 74 |
| 68 | h | 74 | 78 | 257 | b | 71 | 74 |
| 69 | h | 79 | 146 | 258 | b | 71 | 75 |
| 70 | h | 80 | 145 | 259 | b | 72 | 75 |
| 71 | h | 81 | 85 | 260 | b | 72 | 76 |
| 72 | h | 81 | 86 | 261 | b | 74 | 77 |
| 73 | h | 82 | 86 | 262 | b | 74 | 78 |
| 74 | h | 82 | 87 | 263 | b | 78 | 80 |
| 75 | h | 83 | 87 | 264 | b | 79 | 146 |
| 76 | h | 83 | 88 | 265 | b | 80 | 84 |
| 77 | h | 84 | 88 | 266 | b | 80 | 85 |
| 78 | h | 85 | 89 | 267 | b | 80 | 145 |
| 79 | h | 86 | 90 | 268 | b | 80 | 146 |
| 80 | h | 87 | 91 | 269 | b | 81 | 83 |
| 81 | h | 88 | 92 | 270 | b | 81 | 84 |
| 82 | h | 89 | 93 | 271 | b | 82 | 85 |
| 83 | h | 94 | 100 | 272 | b | 82 | 86 |
| 84 | h | 95 | 99 | 273 | b | 82 | 138 |
| 85 | h | 98 | 138 | 274 | b | 83 | 86 |
| 86 | h | 99 | 137 | 275 | b | 83 | 87 |
| 87 | h | 99 | 138 | 276 | b | 84 | 87 |
| 88 | h | 100 | 136 | 277 | b | 85 | 138 |
| 89 | h | 101 | 105 | 278 | b | 85 | 141 |
| 90 | h | 101 | 135 | 279 | b | 85 | 142 |
| 91 | h | 102 | 134 | 280 | b | 85 | 145 |
| 92 | h | 102 | 106 | 281 | b | 85 | 146 |
| 93 | h | 103 | 107 | 282 | b | 86 | 90 |
| 94 | h | 104 | 108 | 283 | b | 86 | 138 |
| 95 | h | 105 | 109 | 284 | b | 87 | 90 |
| 96 | h | 106 | 110 | 285 | b | 88 | 92 |
| 97 | h | 106 | 111 | 286 | b | 88 | 141 |
| 98 | h | 107 | 111 | 287 | b | 89 | 93 |
| 99 | h | 108 | 112 | 288 | b | 89 | 100 |
| 100 | h | 109 | 114 | 289 | b | 89 | 138 |
| 101 | h | 109 | 113 | 290 | b | 89 | 141 |
| 102 | h | 117 | 121 | 291 | b | 91 | 94 |
| 103 | h | 117 | 122 | 292 | b | 91 | 108 |
| 104 | h | 118 | 122 | 293 | b | 92 | 100 |
| 105 | h | 118 | 123 | 294 | b | 92 | 104 |
| 106 | h | 119 | 123 | 295 | b | 92 | 105 |
| 107 | h | 120 | 124 | 296 | b | 92 | 108 |
| 108 | h | 121 | 125 | 297 | b | 92 | 109 |
| 109 | h | 122 | 126 | 298 | b | 92 | 141 |
| 110 | h | 123 | 127 | 299 | b | 93 | 100 |
| 111 | h | 124 | 128 | 300 | b | 94 | 104 |
| 112 | h | 125 | 129 | 301 | b | 94 | 107 |
| 113 | h | 129 | 136 | 302 | b | 94 | 108 |
| 114 | h | 130 | 135 | 303 | b | 95 | 97 |
| 115 | h | 130 | 136 | 304 | b | 97 | 99 |
| 116 | h | 131 | 135 | 305 | b | 97 | 104 |
| 117 | h | 131 | 136 | 306 | b | 97 | 135 |
| 118 | h | 137 | 141 | 307 | b | 99 | 101 |
| 119 | h | 138 | 142 | 308 | b | 99 | 131 |
| 120 | h | 138 | 143 | 309 | b | 99 | 133 |
| 121 | h | 139 | 143 | 310 | b | 99 | 135 |
| 122 | h | 140 | 144 | 311 | b | 99 | 137 |
| 123 | h | 141 | 145 | 312 | b | 100 | 104 |
| 124 | h | 142 | 146 | 313 | b | 100 | 105 |
| 125 | b | 4 | 8 | 314 | b | 100 | 125 |
| 126 | b | 4 | 9 | 315 | b | 100 | 136 |
| 127 | b | 4 | 12 | 316 | b | 100 | 141 |
| 128 | b | 4 | 69 | 317 | b | 101 | 103 |
| 129 | b | 4 | 73 | 318 | b | 101 | 104 |
| 130 | b | 4 | 76 | 319 | b | 101 | 135 |
| 131 | b | 5 | 7 | 320 | b | 102 | 121 |
| 132 | b | 5 | 8 | 321 | b | 102 | 122 |
| 133 | b | 6 | 9 | 322 | b | 102 | 125 |
| 134 | b | 7 | 11 | 323 | b | 105 | 109 |
| 135 | b | 8 | 11 | 324 | b | 105 | 121 |
| 136 | b | 8 | 12 | 325 | b | 105 | 124 |
| 137 | b | 8 | 76 | 326 | b | 105 | 125 |
| 138 | b | 9 | 13 | 327 | b | 106 | 109 |
| 139 | b | 9 | 65 | 328 | b | 106 | 110 |
| 140 | b | 9 | 69 | 329 | b | 106 | 116 |
| 141 | b | 12 | 15 | 330 | b | 106 | 118 |
| 142 | b | 12 | 65 | 331 | b | 106 | 121 |
| 143 | b | 12 | 68 | 332 | b | 107 | 110 |
| 144 | b | 12 | 69 | 333 | b | 107 | 111 |
| 145 | b | 12 | 72 | 334 | b | 108 | 112 |
| 146 | b | 12 | 76 | 335 | b | 109 | 112 |
| 147 | b | 13 | 65 | 336 | b | 109 | 114 |
| 148 | b | 14 | 17 | 337 | b | 109 | 116 |
| 149 | b | 14 | 18 | 338 | b | 109 | 124 |
| 150 | b | 14 | 114 | 339 | b | 110 | 115 |
| 151 | b | 15 | 18 | 340 | b | 114 | 116 |
| 152 | b | 15 | 19 | 341 | b | 116 | 120 |
| 153 | b | 15 | 68 | 342 | b | 116 | 121 |
| 154 | b | 15 | 72 | 343 | b | 116 | 124 |
| 155 | b | 16 | 20 | 344 | b | 117 | 119 |
| 156 | b | 16 | 27 | 345 | b | 117 | 120 |
| 157 | b | 16 | 65 | 346 | b | 118 | 122 |
| 158 | b | 16 | 68 | 347 | b | 119 | 122 |
| 159 | b | 18 | 35 | 348 | b | 119 | 123 |
| 160 | b | 18 | 109 | 349 | b | 119 | 126 |
| 161 | b | 18 | 112 | 350 | b | 121 | 125 |
| 162 | b | 18 | 114 | 351 | b | 122 | 125 |
| 163 | b | 19 | 27 | 352 | b | 122 | 126 |
| 164 | b | 19 | 31 | 353 | b | 123 | 126 |
| 165 | b | 19 | 32 | 354 | b | 123 | 127 |
| 166 | b | 19 | 35 | 355 | b | 124 | 127 |
| 167 | b | 19 | 68 | 356 | b | 125 | 129 |
| 168 | b | 19 | 112 | 357 | b | 125 | 136 |
| 169 | b | 20 | 27 | 358 | b | 126 | 129 |
| 170 | b | 21 | 31 | 359 | b | 127 | 144 |
| 171 | b | 21 | 34 | 360 | b | 128 | 136 |
| 172 | b | 21 | 35 | 361 | b | 128 | 144 |
| 173 | b | 21 | 38 | 362 | b | 130 | 139 |
| 174 | b | 22 | 28 | 363 | b | 130 | 140 |
| 175 | b | 24 | 26 | 364 | b | 130 | 143 |
| 176 | b | 24 | 28 | 365 | b | 131 | 137 |
| 177 | b | 26 | 28 | 366 | b | 133 | 135 |
| 178 | b | 26 | 60 | 367 | b | 136 | 140 |
| 179 | b | 26 | 62 | 368 | b | 136 | 141 |
| 180 | b | 26 | 64 | 369 | b | 136 | 144 |
| 181 | b | 27 | 31 | 370 | b | 137 | 139 |
| 182 | b | 27 | 32 | 371 | b | 137 | 140 |
| 183 | b | 27 | 52 | 372 | b | 138 | 141 |
| 184 | b | 27 | 63 | 373 | b | 138 | 142 |
| 185 | b | 27 | 68 | 374 | b | 139 | 143 |
| 186 | b | 28 | 30 | 375 | b | 141 | 144 |
| 187 | b | 28 | 31 | 376 | b | 141 | 145 |
| 188 | b | 28 | 62 | 377 | b | 142 | 146 |
| 189 | b | 29 | 45 | 378 | b | 143 | 146 |

REFERENCES:

1. Stewart JJP (2007) Optimization of Parameters for Semiempirical Methods V: Modification of NDDO Approximations and Application to 70 Elements. J Mol Model 13: 1173-1213.

2. Mulliken RS (1833) Electronic population analysis on LCAO-MO molecular wave functions. 1. J Chem Phys 23: 1833-1840.

3. R. Ditchfield, W.J. Hehre, Pople JA (1971) Self-Consistent Molecular

Orbital Methods. 9. Extended Gaussian-type basis for molecular-orbital

studies of organic molecules. J Chem Phys 54: 724-728.

4. M. Valiev, E.J. Bylaska, N. Govind, K. Kowalski, T.P. Straatsma, et al. (2010) NWChem: a comprehensive and scalable open-source solution for large scale molecular simulations. Computer Physics Communications 181: 1477-1489.
